# The impact of continued inpatient therapies on functional recovery and community discharge in patients with acquired brain injury

**DOI:** 10.3389/fresc.2026.1804358

**Published:** 2026-04-15

**Authors:** Esha Patel, Aditya Dharani, Simon Gelman, Hayk Petrosyan, Kristen A. Harris

**Affiliations:** 1 Department of Physical Medicine and Rehabilitation, Hackensack Meridian JFK Johnson Rehabilitation Institute, Edison, NJ, United States; 2 Rutgers Robert Wood Johnson Medical School, New Brunswick, NJ, United States; 3 Hackensack Meridian Health Research Institute, Nutley, NJ, United States; 4 Hackensack Meridian School of Medicine, Nutley, NJ, United States

**Keywords:** acquired brain injury (ABI), community discharge, functional outcomes, skilled nursing facility (SNF), traumatic brain injury (TBI), inpatient rehabilitation

## Abstract

**Introduction:**

Inpatient rehabilitation (IRF) is beneficial for patients with acquired brain injuries (ABI), yet some require ongoing therapy at a skilled nursing facility (SNF). While previous research has examined factors influencing community discharge from SNFs, no studies have specifically evaluated ABI patients admitted to SNFs after IRF stays. The aims of this study are to evaluate functional improvements of acquired brain injury patients undergoing rehabilitation at SNF after an IRF stay and determine factors associated with community discharge.

**Methods:**

This is a retrospective cohort study at a SNF unit specialized in brain injury patients and affiliated with an academic medical center. It included patients admitted to a SNF unit from an acute IRF between September 2018 to May 2024, age 18 years or older, with an ABI. The main outcome measures included AM-PAC (Basic Mobility, Daily Activity, and Applied Cognitive) scores and a multivariate analysis of factors associated with community discharge.

**Results:**

Among 218 patients with ABI admitted to SNF, 191 (88%) were discharged to the community and 27 (12%) to long-term care (LTC). Mean AM-PAC scores improved across the entire sample in all subdomains. Patients discharged to LTC had a longer length of stay, time since injury, and lower AM-PAC scores. Admission Basic Mobility scores and a diagnosis of ischemic stroke were predictors of community discharge.

**Discussion:**

Patients with ABI admitted to SNF following an IRF demonstrate significant functional progress, with most patients discharged to the community. Admission functional scores and diagnosis are key factors associated with community discharge.

## 1 Introduction

Acquired brain injury (ABI), including traumatic and non-traumatic injuries, are a major cause of morbidity and mortality in the United States. Acquired brain injuries are common: as per the Centers for Disease Control (1), there were approximately 214,000 traumatic brain injury-related hospitalizations in 2020. Stroke has a higher prevalence: between 2011 and 2022, the self-reported prevalence of stroke in the United States increased by nearly 8% to nearly 800,000 per year (1, 2). Although the epidemiology patterns of different etiologies of ABI vary, patients with ABI share many impairments, including those in activities of daily living (ADLs), motor function, and cognition. As a result, many patients with acquired brain injuries require inpatient rehabilitation after their hospital stay to address new impairments.

Patients admitted to an inpatient rehabilitation facility (IRF) generally require daily medical care by a physician and nursing care. Additionally, they must have a need for at least two therapy disciplines, including physical therapy, occupational therapy, and speech therapy, and the ability to tolerate three hours of therapy per day. It has previously been demonstrated that rehabilitation received in an IRF is associated with lower mortality and better functional outcomes than rehabilitation received in a skilled nursing facility (SNF) or other settings (3, 4). However, over the past several decades, the overall length of stay in IRFs has decreased significantly, including for patients who have suffered severe brain injuries (5, 6). This decrease has been primarily attributed to changes in reimbursement structures for IRFs. Patients with severe ABI often require subsequent admission at a SNF due to persistent impairments (7, 8). This transition ensures they receive the specialized care and support essential for their rehabilitation and recovery.

Recent literature has evaluated various factors associated with successful community discharge from a SNF, demonstrating the significance of demographics and facility characteristics, length of stay, insurer type, health-related variables, and therapy intensity (9–11). In a mixed sample of patients who required a SNF admission following an IRF stay, decreased ability to perform activities of daily living, presence of incontinence, and insurance type were all associated with less successful community discharges (8). Although no studies have specifically evaluated patients with an ABI who required SNF after IRF, a study of TBI patients found that patients with Medicaid enrollment, incontinence, decreased independence with activities of daily living, and cognitive impairment are less likely to discharge to the community from SNF (12). Literature on rates of community discharge from SNF varies with different populations, but it is estimated to be between 43%–54% (13). In patients with brain injuries who present a high level of medical complexity, various additional factors may impact community discharge. Notably, the presence of a tracheostomy tube or a feeding tube, can be associated with less favorable outcomes and may present further care needs (14, 15).

No research has previously been conducted to understand the impact of continued therapy at the SNF level on functional outcomes and rates of community discharge in the acquired brain injury population. This particular group of brain injury patients often presents significant impairments at the time of IRF discharge, which can hinder their transition to community living. The primary objective of this study was to evaluate functional improvements of patients with acquired brain injury undergoing rehabilitation at SNF after an IRF stay. Additionally, the secondary objective of this study was to identify the factors that affect community discharge from SNF in patients with ABIs.

## 2 Methods

### 2.1 General design

This was a retrospective cohort study conducted at a single skilled nursing facility (SNF) that operates independently and is affiliated with an academic medical center. The facility contains a unit dedicated for the care of patients with brain injuries and accepts patients who continue to require therapies at the time of discharge from an IRF. Patients admitted to the SNF unit receive a minimum of two hours of combined physical, occupational, speech, and recreation therapy each day, five days a week. Care is overseen by a brain injury physiatrist, with additional consultants available as needed, including psychiatry and internal medicine. The data were retrospectively extracted from the electronic health records of patients admitted between September 1, 2018, and May 31, 2024. The study was approved by the local Institutional Review Board.

### 2.2 Participants and data collection

Patients were deemed eligible for inclusion in the study if they were aged 18 years or older, had a primary neurologic diagnosis, including stroke, TBI, hypoxic ischemic encephalopathy, brain tumor, or other encephalopathy, and had received rehabilitation at an IRF prior to their admission to the SNF. Those admitted to the SNF directly from an acute care hospital or admitted without a primary brain injury diagnosis were excluded from the study.

Demographic and clinical variables were systematically collected for analysis, including age, gender, race, ethnicity, insurance type, diagnosis, length of stay, time since injury, hospital readmissions, and additional factors that may influence recovery, such as the presence of a tracheostomy and a feeding tube. Discharge disposition was collected for each patient and categorized as either community or long-term care facilities (LTC). Discharges were classified as community if patients were discharged to home, assisted living, or a residential community reentry program.

Functional performance was assessed for each patient by treating therapists at both admission and discharge from the facility. Functional measures included the Activity Measure for Post-Acute Care (AM-PAC) score, which comprises three functional domains: Basic Mobility (BM), Daily Activity (DA), and Applied Cognitive (AC) (16, 17). The AM-PAC is a functional outcomes instrument specifically designed to assess patients’ functional status throughout the continuum of care. It offers a continuous functional score that aligns with real-world capabilities, which are classified into five distinct functional stages for each domain, based on the degree to which a patient can perform various activities (18).

### 2.3 Statistical analysis

Descriptive statistics were utilized to analyze and characterize the data, and continuous variables were summarized by mean (standard deviation) or median (interquartile range), depending on whether the data were normally distributed. The assumption of normality was assessed using the Shapiro–Wilk test of normality. Categorical variables were summarized as frequencies and percentages. Comparison of continuous variables between two groups was conducted using a two-sided t-test or Wilcoxon rank sum test, as appropriate. Categorical variables were compared between groups using two-sided Fisher's exact test or Pearson's chi-square test, as applicable.

A logistic regression model was developed to analyze associations between patients’ characteristics and discharge disposition (community/long-term care). The choice of predictor variables included in the regression models was based on their clinical relevance and results from univariate analyses. The regression results were expressed as odds ratios (ORs) along with 95% confidence intervals (CIs). Model assumptions, multicollinearity, and goodness-of-fit (Hosmer-Lemeshow Go*F* test) were assessed to ensure statistical validity. Unless specified otherwise, any *p*-value <0.05 was considered statistically significant. All data analysis was performed using R software version 4.3.1 (The R Foundation for Statistical Computing, Vienna, Austria) and SAS version 9.4 (19).

## 3 Results

A total of 360 patients were admitted between September 2018 and May 2024. Of 360 patients: 47 patients were excluded because they were admitted directly from acute care or had not yet been discharged at the time of the data collection, 37 patients were discharged to acute care and did not return, and 2 patients became deceased. An additional 54 patients had incomplete charts with missing information, resulting in a total of 218 patients with complete records for this analysis.

Table 1 summarizes the medical and demographic characteristics of the entire sample, stratified by discharge disposition to the community and long-term care (LTC). The sample consisted of 135 (62%) males with an average age of 52 (±15) years, with the most prevalent diagnoses of stroke and traumatic brain injury (TBI). Of the 218 patients, 191 (88%) were discharged to the community, and 27 patients (12%) were discharged to long-term care facilities. The two groups did not differ significantly in age and race distribution (see Table 1); however, findings indicated a higher percentage of female patients discharged to LTC.

**Table 1:** Patient characteristics separated based on discharge to the community vs. long-term care (*n* = 218).

Characteristic	Overall, *N* = 218	Community, *N* = 191	Long Term Care, *N* = 27	*p*-value
Age				0.52^a^
Mean (SD)	52 (15)	52 (15)	50 (15)	
Median (IQR)	54 (41, 63)	55 (41, 63)	53 (39, 61)	
Range	20, 92	20, 92	21, 74	
Gender, *n* (%)				0.12^b^
Female	83 (38%)	69 (36%)	14 (52%)	
Male	135 (62%)	122 (64%)	13 (48%)	
Race, *n* (%)				0.29^c^
Asian	26 (12%)	25 (13%)	1 (3.7%)	
Black	53 (24%)	46 (24%)	7 (26%)	
Hispanic	24 (11%)	23 (12%)	1 (3.7%)	
White	115 (53%)	97 (51%)	18 (67%)	
Diagnosis, *n* (%)				**0**.**008**^c^
Brain Tumor	3 (1.4%)	1 (0.5%)	2 (7.4%)	
Encephalitis/Encephalopathy	14 (6.4%)	13 (6.8%)	1 (3.7%)	
Hemorrhagic stroke	68 (31%)	60 (31%)	8 (30%)	
Hypoxic-ischemic brain injury	22 (10%)	15 (7.9%)	7 (26%)	
Ischemic stroke	47 (22%)	45 (24%)	2 (7.4%)	
Traumatic brain injury	64 (29%)	57 (30%)	7 (26%)	
Length of Stay (days)				**<0**.**001**^a^
Mean (SD)	117 (160)	106 (148)	194 (213)	
Median (IQR)	71 (38, 137)	65 (37, 129)	135 (96, 197)	
Range	5, 1,469	5, 1,469	9, 1,087	
Insurance Groups, *n* (%)				0.48^c^
Dual/Motor Vehicle	14 (6.4%)	12 (6.3%)	2 (7.4%)	
Managed Medicare	14 (6.4%)	12 (6.3%)	2 (7.4%)	
Medicaid	50 (23%)	41 (21%)	9 (33%)	
Medicare	17 (7.8%)	16 (8.4%)	1 (3.7%)	
Commercial	119 (55%)	107 (56%)	12 (44%)	
Worker's compensation	4 (1.8%)	3 (1.6%)	1 (3.7%)	
Tracheostomy, *n* (%)				**<0**.**001**^d^
No	196 (90%)	178 (93%)	18 (67%)	
Yes	22 (10%)	13 (6.8%)	9 (33%)	
Feeding Tube, *n* (%)				**<0**.**001**^b^
No	135 (62%)	129 (68%)	6 (22%)	
Yes	83 (38%)	62 (32%)	21 (78%)	
Time since Injury, (days)				**<0**.**001**^a^
Mean (SD)	69 (53)	64 (47)	104 (76)	
Median (IQR)	53 (38, 77)	49 (37, 74)	77 (57, 117)	
Range	12, 392	12, 392	23, 323	
Multiple admissions, *n* (%)				**0**.**037**^b^
No	171 (78%)	154 (81%)	17 (63%)	
Yes	47 (22%)	37 (19%)	10 (37%)	

Bolded text indicates statistical significance (*p* < 0.05).

^a^ Wilcoxon rank sum test.

^b^ Pearson's Chi-squared test.

^c^ Fisher's Exact Test for Count Data with simulated *p*-value (based on 2000 replicates).

^d^ Fisher's exact test.

The diagnosis distribution showed a significant difference between the two groups (*p* = 0.008). Patients discharged to LTC had a higher proportion of hypoxic-ischemic brain injury and brain tumors. In contrast, ischemic stroke diagnosis was more frequently observed among community-discharged patients. Compared with patients discharged to the community, patients discharged to LTC had a longer average length of stay, longer time since their initial injury/event, higher number of hospital readmissions during their stay, and greater presence of tracheostomies and feeding tubes. Table 2 summarizes patients’ functional status evaluated by AM-PAC scores in three functional domains: Basic Mobility (BM), Daily Activity (DA), and Applied Cognitive (AC) for the entire sample and categorized by discharge disposition. There was a substantial improvement in all three functional domains following SNF admission, resulting in increased scores at discharge compared to the functional assessments at admission (see Table 2).

**Table 2:** Basic mobility, daily activity, and applied cognitive scores on admission and discharge (*N* = 218).

Characteristic	Overall, *N* = 218	Community, *N* = 191	Long Term Care, *N* = 27	*p*-value
Basic Mobility Score Admit				**<0**.**001**^a^
Mean (SD)	26.6 (11.0)	27.9 (10.7)	17.1 (8.5)	
Median (IQR)	30.0 (15.8, 35.5)	31.2 (15.8, 36.4)	11.7 (11.7, 18.1)	
Range	9.8, 58.6	11.7, 58.6	9.8, 38.8	
Basic Mobility Score Discharge				**<0**.**001**^a^
Mean (SD)	37.8 (14.4)	40.0 (12.8)	22.1 (15.5)	
Median (IQR)	39.1 (32.3, 43.9)	39.1 (36.2, 45.3)	11.7 (11.7, 30.2)	
Range	4.2, 78.4	4.2, 78.4	11.2, 63.5	
Daily Activity Score Admit				**<0**.**001**^a^
Mean (SD)	26.7 (11.3)	28.0 (10.7)	17.6 (11.6)	
Median (IQR)	30.9 (17.6, 33.8)	30.9 (21.8, 34.3)	9.7 (9.2, 27.0)	
Range	7.3, 57.3	9.2, 57.3	7.3, 48.5	
Daily Activity Score Discharge				**<0**.**001**^a^
Mean (SD)	35.8 (12.7)	37.6 (11.3)	22.9 (14.5)	
Median (IQR)	36.0 (32.1, 42.8)	36.9 (33.0, 43.5)	17.5 (9.7, 34.1)	
Range	7.8, 66.9	7.8, 66.9	9.2, 56.4	
Applied Cognitive Score Admit				**0**.**011**^a^
Mean (SD)	13.2 (17.3)	14.3 (17.4)	5.1 (13.7)	
Median (IQR)	13.1 (−5.8, 29.2)	19.8 (−5.8, 29.8)	−5.8 (−5.8, 16.3)	
Range	−5.8, 47.5	−5.8, 47.5	−5.8, 33.0	
Applied Cognitive Score Discharge				**<0**.**001**^a^
Mean (SD)	25.1 (16.4)	27.1 (15.2)	11.2 (17.8)	
Median (IQR)	30.0 (20.0, 35.3)	30.7 (24.3, 35.3)	7.5 (−5.8, 28.6)	
Range	−5.9, 58.8	−5.9, 58.8	−5.8, 40.9	

Bolded text indicates statistical significance (*p* < 0.05).

^a^ Wilcoxon rank sum test.

Fisher's Exact Test for Count Data with simulated *p*-value (based on 2000 replicates).

**Figure 1:**
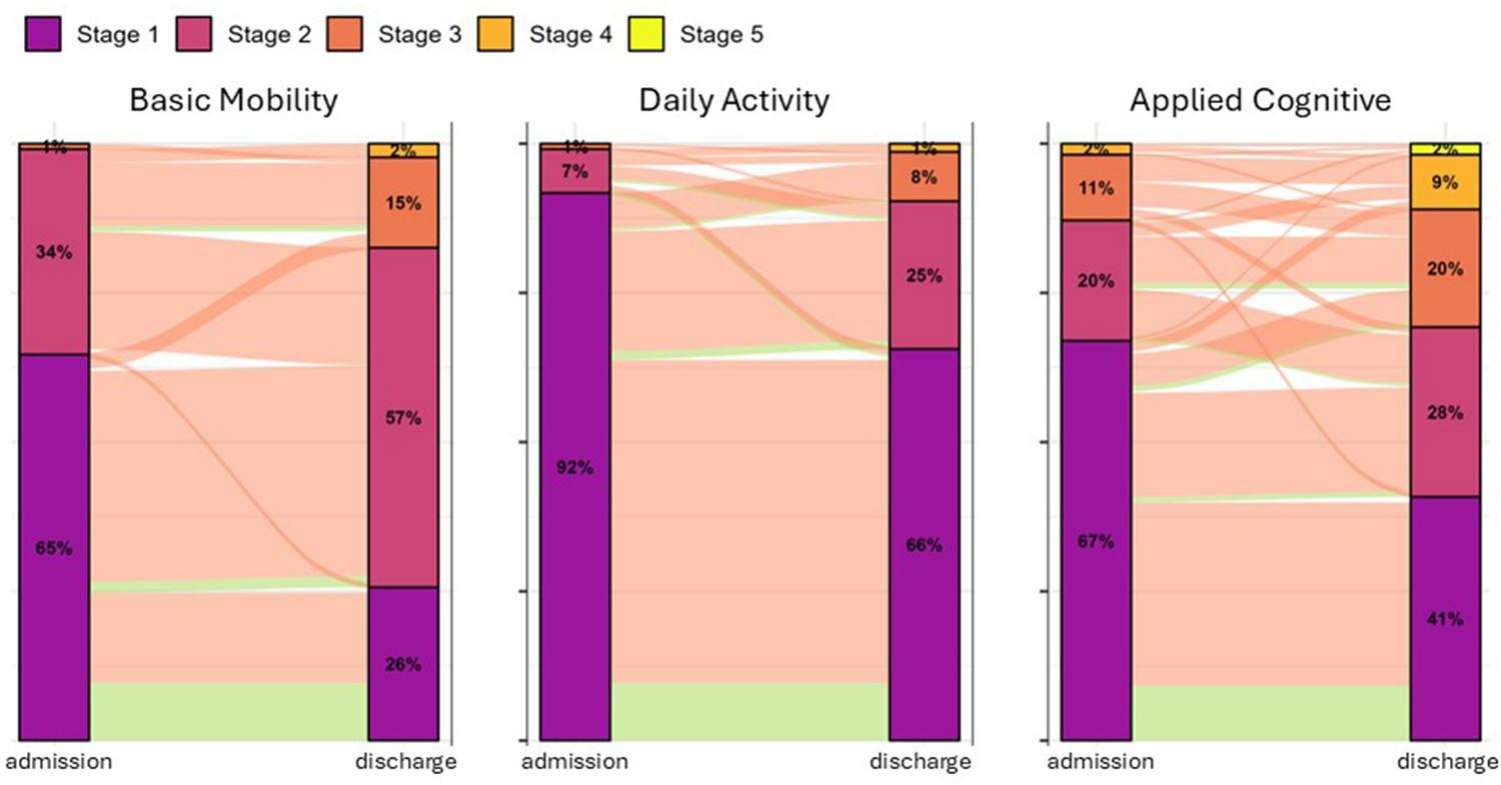
AM-PAC functional stages between admission and discharge. Three alluvial plots demonstrate the shift in AM-PAC functional stages between admission and discharge. For each domain, the vertical bars represent the distribution of patients across five score strata at a single time point: Stage 1 (purple, most limited function), Stage 2 (pink), Stage 3 (dark orange), Stage 4 (light orange), and Stage 5 (yellow, highest function). Percentages inside the bars quantify the proportion of patients in each stage. Semi-transparent ribbons linking the admission and discharge bars trace individual patient transitions, their widths are proportional to the number of patients. Ribbons’ colors (orange and green) identify the patients’ ultimate destinations—community or long-term care, respectively.

A comparison of patients discharged to the community vs. those discharged to LTC facilities showed that individuals discharged to LTC had significantly lower functional abilities at the time of admission compared to their community counterparts (see Table 2). This trend was consistent across all three functional domains. Although both groups demonstrated improvements in functioning during their inpatient stay, the disparity between the community group and the LTC group persisted at discharge. The analysis of AM-PAC functional stages reveals significant improvements in patient functionality after admission to a SNF. At the time of admission, the majority of patients were classified in Stage 1 and Stage 2 across three functional domains. Figure 1 presents alluvial plots for BM, DA, and AC depicting the changes in stages from admission to discharge. Initially, patients experienced “limited movement” for BM domain, “no independent tasks” for DA domain, and “limited life skills” in the AC domain (20). At the time of discharge patients advanced to higher stages, demonstrating enhanced functioning such as “moving around outdoors” for the BM domain, “getting things done” for the DA domain, and experiencing only “minor difficulties” for the AC domain. Supplementary Table S1 compares the percentage of patients among the entire sample in each functional stage at admission and discharge, demonstrating a significant net improvement in functional stages. McNemar–Bowker tests of marginal homogeneity were significant for all three domains, indicating asymmetric admit-to-discharge distributions consistent with net improvement: BM *χ*2 = 111.19 (df = 10), DA *χ*2 = 54.25 (df = 10), and AC *χ*2 = 80.04 (df = 10) (*p* < 0.001).

A logistic regression model was employed to evaluate the factors influencing community discharge in this patient population (Table 3). The model utilized both patient demographics and clinical characteristics. The results revealed that a diagnosis of ischemic stroke and the patient's admission BM score weresignificant predictors of community discharge. Specifically, patients with ischemic stroke had an odds ratio of 50.8 (1.80 to 2,620) for community discharge (*p* = 0.028), while the admission AM-PAC-BM score had an odds ratio of 1.13 (1.02 to 1.26)(*p* = 0.018).

**Table 3:** Association of patient characteristics with a community discharge.

Characteristic	Odds Ratio (95% CI)^a^	*p*-value
Age	0.98 (0.93 to 1.02)	0.30
Gender		
Female	—	
Male	1.78 (0.57 to 5.73)	0.32
Diagnosis		
Brain Tumor	—	
Encephalitis/Encephalopathy	25.2 (0.73 to 1,838)	0.093
Hemorrhagic Stroke	20.4 (0.88 to 814)	0.068
Hypoxic-Ischemic Brain Injury	13.7 (0.44 to 635)	0.14
Ischemic Stroke	50.8 (1.80 to 2,620)	**0**.**028**
Traumatic Brain Injury	17.2 (0.81 to 634)	0.076
Length of Stay	1.00 (1.00 to 1.00)	0.40
Insurance Groups		
Dual/MVA	—	
Managed Medicare	1.18 (0.05 to 27.6)	0.92
Medicaid	0.36 (0.03 to 3.38)	0.41
Medicare	3.41 (0.09 to 450)	0.56
Private	0.97 (0.08 to 7.71)	0.98
Worker's Comp	0.22 (0.01 to 11.8)	0.43
Tracheostomy		
No	—	
Yes	0.50 (0.11 to 2.27)	0.35
Feeding Tube		
No	—	
Yes	0.29 (0.07 to 1.07)	0.071
Time since Injury	1.00 (0.99 to 1.01)	0.76
Multiple admissions		
No	—	
Yes	1.01 (0.30 to 3.66)	0.99
Basic Mobility Score Admit	1.13 (1.02 to 1.26)	**0**.**018**
Daily Activity Score Admit	0.93 (0.82 to 1.05)	0.26
Applied Cognitive Score Admit	1.02 (0.97 to 1.07)	0.43
Basic Mobility Score Gain	1.06 (0.97 to 1.16)	0.24
Daily Activity Score Gain	0.99 (0.89 to 1.09)	0.86
Applied Cognitive Score Gain	1.04 (0.98 to 1.10)	0.23

^a^ CI, confidence interval, bolded text indicates statistical significance (*p* < 0.05).

## 4 Discussion

The aim of this study was to evaluate the functional progress of patients with acquired brain injury admitted to a SNF following inpatient rehabilitation, as well as factors influencing community discharge. Our results demonstrate that patients with ABI continue to experience significant functional improvements during their rehabilitation at the SNF following an IRF admission, and many are successfully transitioning back to the community. Research on the functional gains of patients with ABI admitted to SNF has been limited to date.

A significant number of patients across the entire cohort progressed to higher functional stages according to their AM-PAC scores (20). Notably, even patients discharged to LTC reached a higher functional stage at discharge, which may result in a reduced burden of care in LTC. For instance, a patient progressing in Basic Mobility from Stage 1, “limited movement,” to Stage 2, “limited movement indoors,” may have more independence at their subsequent level of care. Similarly, progression between Applied Cognitive stages may improve communication skills and foster self-advocacy. Importantly, the functional gains observed in this study were greater than the reported minimal clinically important difference (MCID) for patients with ABI found in other studies (20, 21).

More than 88% of patients included in the sample were successfully discharged to the community, including home, assisted living, and dedicated brain injury community re-entry programs. These findings indicate higher rates of community discharge from SNFs than have been reported for the general population. This suggests a potential for further functional improvements and successful community discharge for patients with ABI who are unable to return home following inpatient rehabilitation (13). Patients are transferred from an IRF to a SNF if they are not yet medically or functionally prepared for a safe discharge to their homes. This population often continues to face significant functional impairments. They may also have a need for skilled nursing care, which cannot be adequately provided at home. Furthermore, transfers may be necessary for patients who lack sufficient caregiver support or do not have a safe home environment for discharge to the community. Although it has been widely recognized that patients with ABI demonstrate the most rapid neurological recovery in the first 3–6 months post-injury, research indicates that many patients may remain sensitive to treatment beyond this period (22, 23). In the general SNF population, a study found that only 23.2% of long-stay residents (90–365 days) were discharged to the community (9). Our results also found a correlation between length of stay and discharges to long-term care. However, even within the community discharge population, the median LOS was 63 days with significant variability in the sample. Patients with severe acquired brain injury may require a longer length of stay to achieve community discharge than the general SNF population.

The patient population included in this study is likely more complex than the literature describing the general SNF population, given the known medical complexity of patients with ABI. Nearly half of the sample in this study required a feeding tube, 13% required a tracheostomy, and 23% had a hospital readmission during their SNF stay. Despite these medical complexities, patients showed substantial improvements in all three functional domains of the AM-PAC. Although patients discharged to the community had greater improvements and achieved higher AM-PAC scores at discharge compared to those transferred to long-term care facilities, overall, the findings of this study underscore the potential for continued functional recovery for patients receiving ongoing rehabilitation after IRF discharge.

It is worth noting that this study was conducted within a dedicated brain injury unit at an SNF with trained neurological therapists, as well as a brain injury physiatrist, psychiatrist, and other relevant consultants who may enhance care for this unique patient population. Patients also received at least two hours of therapy daily. Although this study did not specifically evaluate therapy intensity, a recent systematic review found moderate evidence that higher therapy intensity during SNF admission was associated with higher rates of community discharge and shorter LOS (10). Given the unique care and rehabilitation needs of patients with ABI, exploring non-traditional rehabilitation models within the SNF setting could prove beneficial. A previous study of more than 20,000 patients admitted to SNF after stroke identified a dose-response relationship between therapy and community discharge (24). Notably, this correlation was stronger for patients with an uncertain discharge prognosis. These findings are particularly important for patients with ABI, as the likelihood of community discharge often remains unclear early in their rehabilitation process.

Patients with acquired brain injury who require ongoing rehabilitation in the SNF setting are particularly complex and often require services after discharge. In the state where this study was conducted, Medicaid offers additional support and services, including financial assistance for long-term care services. This may have influenced community discharge rates within the patient population examined in this research. A recent study found that increased breadth of Medicaid home and community-based services was associated with a higher likelihood of discharge home from a skilled nursing facility (25). However, a study focusing on the TBI population revealed that Medicaid enrolment, incontinence, decreased independence with activities of daily living, and cognitive impairment were associated with lower odds of successful discharge to the community from SNF (12). Although this study did not find significant differences in insurance type between patients discharged to the community and those discharged to long-term care, further research evaluating the impact of support services available to this patient population is warranted.

### 4.1 Limitations

There are several limitations to this study. Given the retrospective nature of the study, there are some limitations to the data, including missing AM-PAC scores for some of the earliest patients admitted. Information on factors preceding the skilled nursing facility admission, such as acute care and inpatient rehabilitation length of stay, was unavailable. Additionally, there were several other unmeasured confounders, including behavioural and neuropsychiatric disturbances, home environment, and the presence of family support. Furthermore, the generalizability of this study may be limited as not all SNF units operate under the same model, particularly in regards to therapy intensity. The analysis also did not assess the amount of therapy received and its possible impact on outcomes. Additionally, the heterogeneous nature of the sample may limit the statistical power and generalizability of the findings. Nevertheless, the data suggest that complex ABI patients may benefit from continued neurologic rehabilitation in the SNF setting when discharge home from an IRF is not feasible.

### 4.2 Conclusions

Continued rehabilitation at a skilled nursing facility after admission to an inpatient rehabilitation facility is beneficial for patients with severe acquired brain injury and leads to substantial functional improvements. Patients admitted to a SNF following an IRF stay can demonstrate progress across all AM-PAC domains, with most patients able to be discharged to the community after their rehabilitation stay.

## Data Availability

The raw data supporting the conclusions of this article will be made available by the authors, without undue reservation.
