# Supplementary material for: The impact of continued inpatient therapies on functional recovery and community discharge in patients with acquired brain injury
Source: Front Rehabil Sci. 2026 Apr 15;7:1804358. doi: 10.3389/fresc.2026.1804358 (PMC13125107; doi:10.3389/fresc.2026.1804358)
Supplement: Supplementary file 1 [file Table1.docx]

Supplementary Material

**Supplementary Table 1:** Comparison of Admission and Discharge AM-PAC Stages Across All Domains.

| **Admission AM-PAC Stage** | **Stage 1** | **Stage 2** | **Stage 3** | **Stage 4** | **Stage 5** |
| --- | --- | --- | --- | --- | --- |
| **Basic Mobility** | | | | | |
| **Stage 1** | 54 (24.8%) | 81 (37.2%) | 6 (2.8%) | 0 (0.0%) | 0 (0.0%) |
| **Stage 2** | 2 (0.9%) | 43 (19.7%) | 25 (11.5%) | 5 (2.3%) | 0 (0.0%) |
| **Stage 3** | 0 (0.0%) | 0 (0.0%) | 2 (0.9%) | 0 (0.0%) | 0 (0.0%) |
| **Stage 4** | 0 (0.0%) | 0 (0.0%) | 0 (0.0%) | 0 (0.0%) | 0 (0.0%) |
| **Stage 5** | 0 (0.0%) | 0 (0.0%) | 0 (0.0%) | 0 (0.0%) | 0 (0.0%) |
| **Daily Activity** | | | | | |
| **Stage 1** | 139 (63.8%) | 47 (21.6%) | 14 (6.4%) | 0 (0.0%) | 0 (0.0%) |
| **Stage 2** | 4 (1.8%) | 6 (2.8%) | 3 (1.4%) | 3 (1.4%) | 0 (0.0%) |
| **Stage 3** | 0 (0.0%) | 1 (0.5%) | 1 (0.5%) | 0 (0.0%) | 0 (0.0%) |
| **Stage 4** | 0 (0.0%) | 0 (0.0%) | 0 (0.0%) | 0 (0.0%) | 0 (0.0%) |
| **Stage 5** | 0 (0.0%) | 0 (0.0%) | 0 (0.0%) | 0 (0.0%) | 0 (0.0%) |
| **Applied Cognitive** | | | | | |
| **Stage 1** | 87 (39.9%) | 40 (18.3%) | 14 (6.4%) | 4 (1.8%) | 1 (0.5%) |
| **Stage 2** | 0 (0.0%) | 19 (8.7%) | 19 (8.7%) | 5 (2.3%) | 1 (0.5%) |
| **Stage 3** | 2 (0.9%) | 3 (1.4%) | 9 (4.1%) | 9 (4.1%) | 1 (0.5%) |
| **Stage 4** | 0 (0.0%) | 0 (0.0%) | 1 (0.5%) | 2 (0.9%) | 1 (0.5%) |
| **Stage 5** | 0 (0.0%) | 0 (0.0%) | 0 (0.0%) | 0 (0.0%) | 0 (0.0%) |
